# Supplementary material for: Multimodal thrombectomy device for treatment of acute deep venous thrombosis
Source: Sci Rep. 2022 Mar 28;12:5295. doi: 10.1038/s41598-022-09001-6 (PMC8964697; doi:10.1038/s41598-022-09001-6)
Supplement: Supplementary file 1 — Supplementary Information. [file 41598_2022_9001_MOESM1_ESM.pdf]

Supplement

Mechanics model for vein wall distention and collapse

To study the relationship between a blood vessel volume and pressure, we analyzed the model problem of a hyperelastic cylinder subjected to internal pressure. The vena cava was modeled as a right cylinder of initial wall thickness H and radius R. When inflated, the cylinder retains its shape but changes it thickness to h and radius to r. The model assumed that the cylinder wall does not rupture and treated the wall as an incompressible, isotropic, Neo-Hookean elastic material. Due to the occlusion of the treatment zone by the distal and proximal balloons, which were connected by a relatively stiff catheter, the length of the cylinder was held constant. Shown in figure 1, the basis of $\left\{\hat{e}_r, \quad \hat{e}_\theta, \quad \hat{e}_z\right\}$,

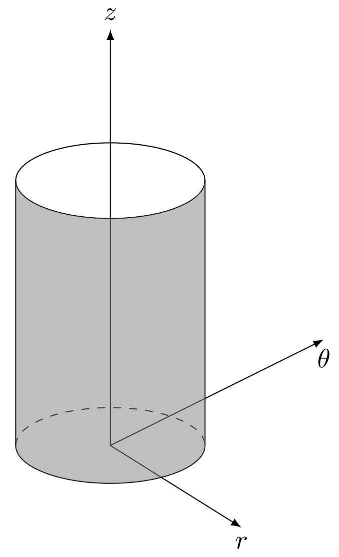

Figure 1: Cylindrical coordinate basis and cylindrical representation of the vena cava.

or the cylindrical coordinate system, will be used.

Kinematics

The first step was to relate the changes in the radius of the cylinder to the degree that the material in the cylinder wall stretches. In examining a cross-sectional slice of the hollow cylinder, the three principal stretches (λ_r, λ_θ, and λ_z) needed to compose the deformation gradient tensor can be written, where a principal stretch is defined as the ratio of the deformed length of a piece of material in one of the three coordinate directions (r, θ, or z) to the initial length of that piece of material in that coordinate direction.

λ_r, the stretch ratio in the radial direction, is the ratio of initial to final wall thickness:

$$\lambda_r = \frac{h}{H}. \tag{1}$$

λ_θ, the stretch ratio in the "hoop" (circumferential) direction, is the ratio of final to initial circumference:

$$\lambda_\theta = \frac{2\pi r}{2\pi R} = \frac{r}{R} \tag{2}$$

Finally, the stretch λ_z along the axis of the cylinder is unity because the length of the treatment zone is fixed by the connection between the proximal and distal balloons, the final length l and initial length L are identical, so that:

$$\lambda_z = \frac{l}{L} = 1 \tag{3}$$

To enforce the constraint that the wall of the vena cava as incompressible, we note that the initial and final volume are $V = 2\pi RLH = 2\pi rlh$, so that

$$\frac{2\pi rlh}{2\pi RLH} = \lambda_r \lambda_\theta = 1, \tag{4}$$

or $\lambda_r = \lambda_\theta^{-1}$.

Stresses

The next step is to examine the three stresses in the wall of the cylinder: σ_r, σ_θ, and σ_z. Because the thickness of the cylinder is significantly smaller than its radius, σ_r can be neglected relative to the other terms so that the stress state is approximately two-dimensional. These stresses can be calculated from the "Law of Laplace" by treating the cylinder as a membrane in equilibrium with an internal pressure, p, that arises from fluid inside the vena cava or the thrombus itself, and acts uniformly on the internal lumen and both ends of the vena cava. Considering the equilibrium of a cross-section of the vena cava, the a stress σ_θ, acting over the circumferential cross-sectional area $2\pi rh$, must balance the internal pressure acting over an area πr^2, so that $2\pi rh\sigma_z = \pi r^2 p$, or:

$$\sigma_z = \frac{pr}{2h} \tag{5}$$

A second force balance can be achieved by examining the cylinder if it were cut in half lengthwise. In this case, p acts over an area $2rl$, and is counteracted by σ_θ which acts on the membrane walls of area $2hl$, so that $\sigma_\theta hl = prl$, or:

$$\sigma_\theta = \frac{pr}{h} \tag{6}$$

Constitutive Behavior

Finally, the constitutive relation that relates stress to stretch of the vena cava was considered. The vena cava was modeled as isotropic and neo-Hookean, so that:

$$\sigma_\theta = 2\mu J^{-5/3}(\lambda_\theta^2 - \lambda_r^2) \tag{7}$$

$$\sigma_z = 2\mu J^{-5/3}(\lambda_z^2 - \lambda_r^2) = 2\mu J^{-5/3}(1 - \lambda_r^2) \tag{8}$$

where $J = \lambda_r \lambda_\theta \lambda_z = \lambda_r \lambda_\theta$. Substituting:

$$\frac{pr}{h} = 2\mu J^{-5/3}(\lambda_\theta^2 - \lambda_r^2) \tag{9}$$

$$\frac{pr}{2h} = 2\mu J^{-5/3}(1 - \lambda_r^2) \tag{10}$$

Solving:

$$1 - \lambda_r^2 = \lambda_\theta^2 - 1 \tag{11}$$

Substituting once more:

$$\frac{pr}{h} = p\frac{\lambda_\theta R}{\lambda_r H} = 2\mu J^{-5/3}(2\lambda_\theta^2 - 2) \tag{12}$$

$$p = 2\mu \frac{\lambda_r H}{\lambda_\theta R} \frac{(2\lambda_\theta^2 - 2)}{(\lambda_r \lambda_\theta)^{5/3}} \tag{13}$$

$$= 4\mu \frac{H}{R} \frac{(\lambda_\theta^2 - 1)}{(\lambda_r^2 \lambda_\theta^8)^{1/3}} \tag{14}$$

$$= 4\mu \frac{H}{R} \frac{(\lambda_\theta^2 - 1)}{(2\lambda_\theta^8 - \lambda_\theta^{10})^{1/3}} \tag{15}$$
